# Temporal and spatial distribution characteristics in the natural plague foci of Chinese Mongolian gerbils based on spatial autocorrelation

**DOI:** 10.1186/s40249-017-0338-7

**Published:** 2017-08-07

**Authors:** Hai-Wen Du, Yong Wang, Da-Fang Zhuang, Xiao-San Jiang

**Affiliations:** 1 State Key Laboratory of Resources and Environmental Information Systems, Institute of Geographical Sciences and Natural Resources Research, Chinese Academy of Sciences, 11A Datun Road, Chaoyang District, Beijing, 100101 China; 2 College of Resources and Environmental Science, Nanjing Agricultural University, No.6 Tongwei Road, Nangjing, 210095 China

**Keywords:** Geographic information system, Temporal and spatial distribution, Spatial autocorrelation, Moran’s I, Body fleas, Plague natural focus of Mongolian gerbils, China

## Abstract

**Background:**

The nest flea index of *Meriones unguiculatus* is a critical indicator for the prevention and control of plague, which can be used not only to detect the spatial and temporal distributions of *Meriones unguiculatus*, but also to reveal its cluster rule. This research detected the temporal and spatial distribution characteristics of the plague natural foci of Mongolian gerbils by body flea index from 2005 to 2014, in order to predict plague outbreaks.

**Methods:**

Global spatial autocorrelation was used to describe the entire spatial distribution pattern of the body flea index in the natural plague foci of typical Chinese Mongolian gerbils. Cluster and outlier analysis and hot spot analysis were also used to detect the intensity of clusters based on geographic information system methods. The quantity of *M. unguiculatus* nest fleas in the sentinel surveillance sites from 2005 to 2014 and host density data of the study area from 2005 to 2010 used in this study were provided by Chinese Center for Disease Control and Prevention.

**Results:**

The epidemic focus regions of the Mongolian gerbils remain the same as the hot spot regions relating to the body flea index. High clustering areas possess a similar pattern as the distribution pattern of the body flea index indicating that the transmission risk of plague is relatively high. In terms of time series, the area of the epidemic focus gradually increased from 2005 to 2007, declined rapidly in 2008 and 2009, and then decreased slowly and began trending towards stability from 2009 to 2014. For the spatial change, the epidemic focus regions began moving northward from the southwest epidemic focus of the Mongolian gerbils from 2005 to 2007, and then moved from north to south in 2007 and 2008.

**Conclusions:**

The body flea index of Chinese gerbil foci reveals significant spatial and temporal aggregation characteristics through the employing of spatial autocorrelation. The diversity of temporary and spatial distribution is mainly affected by seasonal variation, the human activity and natural factors.

**Electronic supplementary material:**

The online version of this article (doi:10.1186/s40249-017-0338-7) contains supplementary material, which is available to authorized users.

## Multilingual abstracts

Please see Additional file 1 for translations of the abstract into the five official working languages of the United Nations.

## Background

Plague is a zoonotic disease caused by the bacillus *Yersinia pestis*. Plague bacteria circulate mainly in rodent hosts and are transmitted between them and to other mammals via adult fleas, and predation or cannibalism [1, 2]. Fleas play a major role in maintaining the natural foci of the plague epidemic [3]. Plague is an infectious disease transmitted by infected fleas that has caused large epidemics around the world and resulted in thousands of people dying. Despite advances in understanding the biology, epidemiology and control of this disease, plague is far from being eradicated [4].

Plague has been reported in the epidemic foci of Mongolian gerbils in China over the last ten years. The plague has broken out in the Ningxia Hui Autonomous Region in 2005, 2006, 2009, and 2013, and also broke out in Dingbian County, Shaanxi Province in 2005 and in Kangbao County, Hebei Province in 2002, 2003, and 2005. Plague broke out in the Inner Mongolia Autonomous Region from 2004 to 2011, and in 2014. Nine episodes of human plague were confirmed in 6 years from 1970 to 2004 (1970, 1972, 1986, 1987, 1991 and 2004) in the seven counties of the Inner Mongolia Autonomous Region. These cases occurred within the plague foci of the Mongolian gerbil. Seven of the nine patients (77.78%) became infected via a flea bite in the wild.

In order to better prevent and control the epidemic of plague, this study aims to strengthen the existing research on body fleas index in the natural focus of plague relating to typical Chinese Mongolian gerbils. Body flea is an important transmission media for plague that can be used to analyze the temporal and spatial distribution in the epidemic foci of Mongolian gerbils. Some studies showed that the transmission of animal plague is caused by the interactions of *Meriones unguiculatus* with its flea vectors [5, 6].

A geographic information system (GIS) is an invaluable aid in measuring the spatial distribution of a disease. GIS have been used to analyze epidemiology and public health problems, and they provide strong technical support in utilizing ecological methodology, realizing temporal analyses, and aid in scientific decision-making [7]. Establishing a database of animal hosts of the plague by analyzing the rule of the spatiotemporal distribution and characteristics in the plague foci of China is required [8]. Ecological niche modeling has been used to predict the potential distribution of the Himalayan marmot plague natural epidemic foci in China [9–11]. GIS has also been used to predict the risk of plague across the western US by modeling the ecologic niche of plague [12]. The burrows of great gerbils were analyzed by point pattern analysis of spatial distribution patterns in Kazakhstan [13]. *Amphipsylla* fleas and rodent hosts were identified with the geographical range sizes of ectoparasites [14]. The *Yersinia pestis* ecotype was analyzed using a spatial analysis, which resulted in a habitat-suitability model and the creation of a plague risk map to confirm areas that are dangerous to humans [15]. A mathematical compartment model was used to describe the geographical and temporal spread of an epidemic of pneumonic plague [16]. The global modeling technique which can reveal single and multivariate observational time series has been applied to analyze the plague epidemic data in Bombay from 1896 to 1911 [17].

Spatial epidemiology is now more of a necessity for outbreak investigations, surveillance, hypothesis testing, and generating follow-up activities necessary to perform a complete and proper epidemiologic analysis [18]. It is important that those engaged in all aspects of public health surveillance are aware of the distribution and epidemiology of this group of diseases and can prepare for their control when necessary [19]. Thus far, it has been uncommon to use spatial autocorrelation in a GIS for predicting plague outbreaks.

This study analyzed the distribution characteristics of the body flea index relating to Chinese Mongolian gerbils in the natural foci of plague during different periods and spatial areas. Furthermore, it explored the hot spot regions relating to the body flea index during different periods.

## Methods

### Study region

**Fig. 1:**
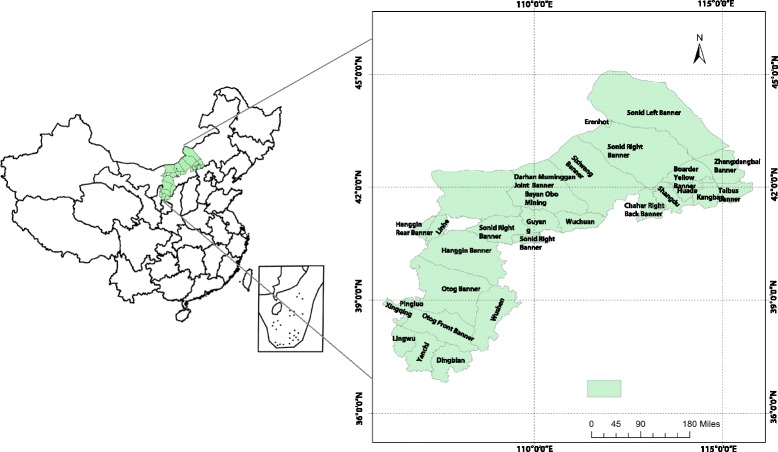
Location of the study area Map of the study area divided into 29 counties (district, banner, and city).

The epidemic foci of the Mongolian gerbil cover an area of roughly 100,000 km^2^; the epidemic foci area is large and extensively affected (see Fig. 1). The foci include Bayannur City, Ordos City, Baotou City, Hohhot City, Ulanqab City, and 24 banners (counties, cities, districts) governed by the Xilingol League in the western area of the Inner Mongolia Autonomous Region; Dingbian County in Shaanxi Province; Xingqing District of Yinchuan City, Lingwu City, Pingluo County, and Yanchi County in the Ningxia Hui Autonomous Region; and Kangbao County in Hebei Province.

The boundary of the epidemic focus of the Mongolian gerbil between the Ulanqab Plateau and the Ordos Plateau is the Huanghe (Yellow River). The Ulanqab Plateau belongs to the mesothermal desert steppe; the terrain slopes gently with a vast territory and a higher distribution trend of terrain has been noticed in the south than in the north. The Ulanqab Plateau is the oldest land mass that is surrounded by the Huanghe River; the plateau is in the northern area of the Loess Plateau, which is characterized by warm temperate desert grassland. This part of the foci is relatively independent from the other regions, using the Huanghe River as the geographical barrier to the northwest of the foci, the Loess Plateau to the south, and the Kubuqi sandy-land and Mu Us desert sands. On the east of the foci, there are low amounts of precipitation and over-evaporation; a serious shortage of surface water and groundwater exists, caused by an ever-increasing share of human activity seriously destroying the environment. Consequently, the Aeolian landform has developed into a sand deposit and has become a more suitable area for rodents to live. The epidemic foci of the Mongolian gerbil are located in the northern region, where is continental climate. These environments provide suitable habitat conditions for plague prevalence among rats; the foci have also become the natural focus of plague relating to typical Chinese Mongolian gerbils.

### Data sources and processing methods

The Mongolian gerbil body fleas were under investigation from April to July and then from October to November, from 2005 to 2014 (Table 1 shows the regions of data collection and the years). Monthly data were merged into yearly data. Then, the annual average body flea index was calculated to obtain the effective body flea index data from 2005 to 2014.

**Table 1:** Body flea index used in the study, by region, from 2005 to 2014

Regions of data collection	Year(s)
Bayan Obo Mining District	2007, 2008, 2009, 2010, 2011, 2012, 2013, 2014
Border Yellow Banner	2011, 2012, 2013, 2014
Chahar Right Back Banner	2010, 2011, 2012, 2013, 2014
Darhan Muminggan Joint Banner	2007, 2008, 2009, 2010, 2011, 2012, 2013, 2014
Dingbian County	2005, 2006, 2007, 2008, 2009, 2010, 2011, 2012, 2013, 2014
Erenhot city	2005, 2006, 2007, 2008, 2009, 2010, 2011, 2012, 2013, 2014
Guyang County	2010, 2011, 2012, 2013, 2014
Hanggin Banner	2014
Hondlon District	2007, 2008, 2009
Huade County	2009, 2010, 2011, 2012, 2013, 2014
Jiuyuan District	2008, 2009, 2010, 2011, 2012, 2013, 2014
Kangbao County	2005, 2006, 2007, 2008, 2009, 2010, 2011, 2012, 2013, 2014
Lingwu city	2005, 2006, 2007, 2008, 2009, 2010, 2011, 2012, 2013, 2014
Otog Banner	2006, 2007, 2009, 2010, 2012, 2013, 2014
Otog Front Banner	2007, 2008, 2009, 2010, 2011, 2012, 2013, 2014
Pingluo County	2008, 2009, 2010, 2011, 2012, 2013, 2014
Shangdu County	2009, 2010, 2011, 2012, 2013, 2014
Siziwang Banner	2009, 2010, 2011, 2012, 2013, 2014
Sonid Left Banner	2005, 2007, 2008, 2009, 2010, 2011, 2012, 2013, 2014
Sonid Right Banner	2005, 2006, 2007, 2008, 2009, 2010, 2011, 2012, 2013, 2014
Taibus Banner	2007, 2012, 2013, 2014
Urad Front Banner	2007, 2008, 2009, 2010, 2011, 2012, 2013, 2014
Urad Middle Banner	2005, 2006, 2007, 2008, 2009, 2010, 2011, 2012, 2013, 2014
Wuchuan County	2006, 2007, 2008, 2009, 2010, 2011, 2012, 2013, 2014
Wusheng County	2005, 2006, 2007, 2008, 2009, 2010, 2011, 2012, 2013, 2014
Xingqing District	2006, 2007, 2008, 2009, 2010, 2011, 2012, 2013, 2014
Yanchi County	2005, 2006, 2007, 2008, 2009, 2010, 2011, 2012, 2013, 2014
Zhengxiangbai Banner	2005, 2006, 2007, 2008, 2009, 2010, 2011, 2012, 2013, 2014

The calculation formula is as follows:

1 $$ BFI=\frac{TNF}{TNM}, $$

where BFI is the value of the body flea index, TNF is the total number of fleas, and TNM is the total number of Mongolian gerbils.

**Fig. 2:**
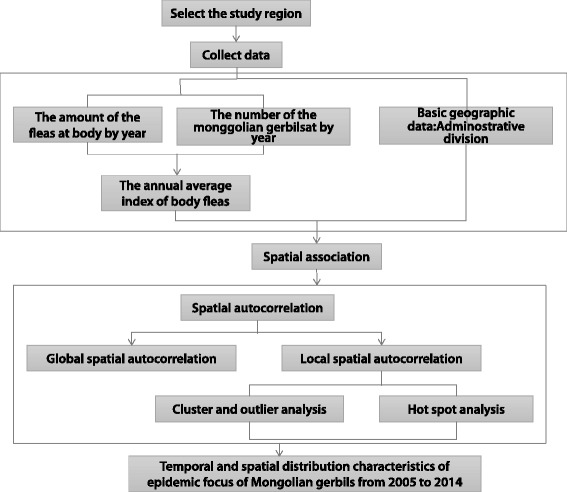
Technical analysis flowchart

Data on the Mongolian gerbils’ body fleas were collected from local surveillance sites of the plague in the epidemic foci of the Mongolian gerbils. The epidemic foci of the Mongolian gerbils’ study area map were extracted from the Chinese Academy of Sciences Resources and the Environment Scientific Information Center and it also provided 1:10,000 scale Chinese county electronic maps. A global means of spatial autocorrelation was used with Moran’s I to describe the entire spatial distribution pattern of the body fleas index computed using ArcGIS10.2, through local means of spatial autocorrelation with cluster and outlier analysis (Anselin Local Moran’s I) and hot spot analysis (Getis-Ord ${G}_{\mathrm{i}}^{\ast }$) to achieve the temporal and spatial distribution characteristics in plague natural focus of typical Chinese Mongolian gerbils (see Fig. 2).

### Research technique

Spatial autocorrelation analysis was used as a statistical method of spatial statistics to test the attribute value of an element as to whether it was dramatically associated with its adjacent unit. This analysis method can classify into positive correlation and negative correlation; positive correlation indicates that the attribute value of a spatial unit and its adjacent unit have the same change tendency, while negative correlation indicates the opposite [20]. Spatial autocorrelation includes global and local spatial autocorrelation [21]. Local spatial autocorrelation is the necessary component for global spatial autocorrelation, and global spatial autocorrelation analyzes the homogeneity domain and the whole regularity of the phenomena distribution. Local spatial autocorrelation analyzes the heterogeneity domain and finds the concealed abnormal value, unlike what the global spatial autocorrelation finds. The presupposition of spatial autocorrelation is that a quantitative expression of the spatial relation between elements of geographical areas is given by the spatial weight matrix through the inverse distance of the automatically generated spatial weight data required in the analysis.

Global spatial autocorrelation: This section addresses spatial autocorrelation based on the distribution of the body fleas index using Global Moran’s I statistic. The spatial autocorrelation tool evaluates whether the pattern expressed is clustered, dispersed, or random.

The formula is as follows:

2 $$ I=\frac{n\bullet \sum_{i=1}^n\sum_{j=1}^n{\omega}_{ij}\left({x}_i-x\right)\left({x}_j-x\right)}{\left(\sum_{i=1}^n\sum_{j=1}^n{\omega}_{ij}\right)\bullet \sum_{i=1}^n{\left({x}_i-x\right)}^2},\kern0.5em i\ne j, $$

where *x*_*i*_ and *x*_*j*_ are the observed values of the variable *x* at the *i* and *j* regions, respectively;*ω*_*ij*_ is a binary weighting matrix for the adjacent spaces; *i* = 1,2,3,…,*n*, and *j* = 1,2,3,…,*n*. When the regions *i* and *j* are adjacent, *ω*_*ij*_ = 1; otherwise, *ω*_*ij*_ = 0. The range of Moran’s I value is [−1, 1].

The spatial autocorrelation tool calculates the *z*-score and the expected index, and the variance is calculated as:

3 $$ {Z}_I=\frac{I-\left(\frac{-1}{n-1}\right)}{\sqrt{\left(E\left[{I}^2\right]-E{\left[I\right]}^2\right)}}, $$

where the *z*-score or *P*-value indicate a statistical significance, a positive Moran’s I index value indicates a tendency toward clustering, while a negative Moran’s I index value indicates a tendency towards dispersion; zero indicates a tendency towards random.

Cluster and outlier analysis: Anselin Local Moran’s I of local statistics can judge whether the spatial clustering pattern of the body flea index is high or low. The formula is:

4 $$ {I}_i=\frac{x_i-x}{\frac{\sum_{j=1,j\ne 1}^n{\left({x}_i-x\right)}^2}{n-1}-{x}^2}\sum_{j=1,j\ne 1}^n{\omega}_{i,j}\left({x}_j-x\right) $$

The *z*-score formula is:

5 $$ Z\left({I}_i\right)=\frac{I_i-E\left({\mathrm{I}}_i\right)}{\sqrt{VAR\left({I}_i\right)}}, $$

where *ω*_*ij*_ is a binary weighting matrix for adjacent spaces. A positive *I*_*i*_ indicates a spatial clustering of similar values surrounding the unit space *i*, and a negative *I*_*i*_ indicates a spatial clustering of non-similar values surrounding the unit *i*.

A high positive *z*-score can be noticed as well as the similar values surrounding this area (either high or low) [22]. A statistically significant cluster region of high values will be represented with high-high (HH) and low-low will indicate a statistically significant cluster of low values. A low negative *z*-score for a feature indicates a statistically significant spatial data outlier. The feature class will indicate if the feature has a high value and is surrounded by features with low values (HL) or if the feature has a low value and is surrounded by features with high values (LH).

Hot spot analysis: Getis-Ord ${G}_{\mathrm{i}}^{\ast }$ [23] is an index used to estimate the inner spatial heterogeneity of an area, to recognize the distribution of hot spot regions and cold spot regions in different areas, and to reflect the correlation extent of the unit attributive values between a certain region and a region close to a certain region by analyzing the information in the sub-area.

6 $$ {G}_i^{\ast }=\frac{\sum_{j=1}^n{\omega}_{i,j}{x}_j-x\sum_{j=1}^n{\omega}_{i,j}}{\sqrt{\frac{\sum_{j=1}^n{x}_j^2}{n}-{x}^2}\sqrt{\frac{\left[n\sum_{j=1}^n{\omega_i^2}_{,j}-{\left(\sum_{j=1}^n{\omega}_{i,j}\right)}^2\right]}{n-1}}}, $$

where *ω*_*ij*_ is a binary weighting matrix for the adjacent spaces. A high *z*-score and small *P*-value for a feature indicates a spatial clustering of high values. A low negative *z*-score and small *P*-value indicates a spatial clustering of low values. The higher (or lower) the *z*-score, the more intense the clustering is. A *z*-score near zero indicates no apparent spatial clustering.

## Results

### Global spatial autocorrelation

The Global Moran’s I results of the body flea index from 2005 to 2014 were 0.2442, 0.0121, 0.1121, −0.1062, 0.1387, −0.0844, −0.1778, 0.0197, 0.1409, and 0.0044 (see Table 2), respectively, for each year. The Global Moran’s I values were greater than zero in 2005, 2006, 2007, 2009, 2012, 2013, and 2014. The *z*-scores were greater than 1.96 (2.3019, 2.1963) in 2005 and 2009; the *P*-values are significant at 0.05 or above, which indicates that the body flea index was spatially clustered in 2005 and 2009. These results demonstrate that the body flea index in the epidemic foci of Mongolian gerbils agglomerated spatially with relatively higher levels in 2005 but lower levels in 2009; the body flea index was randomly spatial in other years.

**Table 2:** Result of the general autocorrelation analysis on the distribution of body flea index

Year	Moran’s I	Expected I	Variance	*z*-score	*P*-value	Result
2005	0.2442	−0.0345	0.0147	2.3019	0.0213	clustered
2006	0.0121	−0.0345	0.0074	0.5432	0.5870	random
2007	0.1121	−0.0345	0.0087	1.5666	0.1172	random
2008	−0.1062	−0.0345	0.0126	−0.6401	0.5221	random
2009	0.1387	−0.0345	0.0062	2.1963	0.0281	clustered
2010	−0.0844	−0.0345	0.0099	−0.5022	0.6155	random
2011	−0.1778	−0.0345	0.0100	−1.4331	0.1518	random
2012	0.0197	−0.0345	0.0137	0.4619	0.6442	random
2013	0.1409	−0.0345	0.0134	1.5165	0.1294	random
2014	0.0044	−0.0345	0.0155	0.3120	0.7550	random

### Local spatial autocorrelation

#### Cluster and outlier analysis

**Fig. 3:**
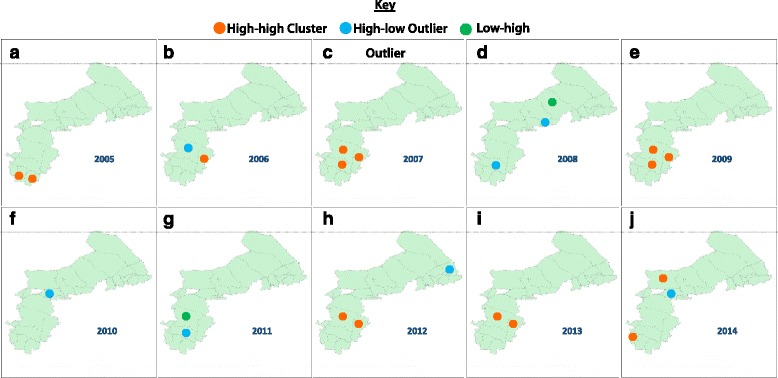
Cluster analysis using Anselin local Moran’s I, the results of spatial distribution patterns of the body flea index in the period from 2005 to 2014 (**a**–**j**)

Using Anselin Local Moran’s I, the distribution patterns of the body flea index were obtained; meanwhile, the maps show the cluster and outlier locations (see Fig. 3). There are three distribution patterns of the body flea index, namely high-high (HH), high-low (HL), and low-high (LH). The primary distribution mode of the area of body flea index represents HH which means relative high body flea index being observed in epidemic focus of plague. The outlier (HL, LH) distribution only found in some districts and the features of HL region and the LH region are individually representing two different situations: The value of the body flea index in these districts was higher than that of the surrounding areas, and the value of the body flea index in these districts was higher than that of the surrounding areas. Thus, we can infer that these differences are related to the geographical environment characteristics. However, when compared with the surrounding areas and their geographical environment characteristics, the value of the body flea index of these districts was lower than in the surrounding areas. Further study is needed to explore the environmental growth and decline of body fleas in these districts of outlier distribution patterns. The body flea index of other districts showed no statistically significant data and random distributions.

The important years in which the body flea index changed were 2005, 2006, 2009, 2013, and 2014. Further studies are required on the environmental growth and decline of body fleas in these years, especially in Otog Banner and Wusheng County where patterns of the body flea index changed.

#### Hot spot analysis

**Fig. 4:**
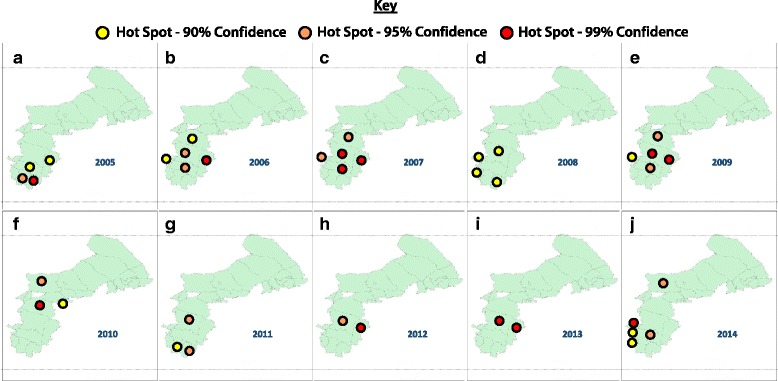
Hot spot analysis using Getis-Ord ${G}_{\mathrm{i}}^{\ast }$, the results of hot spot regions of the body flea index in the period from 2005 to 2014 (**a**–**j**)

The Getis-Ord ${G}_{\mathrm{i}}^{\ast }$ index of the body flea index was comparatively high and the epidemic mainly centered in the southwest from 2005 to 2014 (see Fig. 4). There were hot spot regions in terms of the body flea index in 2006, 2007, and 2009 (Wusheng County, Hanggin Banner, Pingluo County, Otog Front Banner, and Otog Banner) and in 2014 (Urad Middle Banner, Lingwu city, Xingqing District, Pingluo County, and Otog Front Banner), in which the percentage of positive hot spot regions to the total area was 18.52%. The hot spot regions in terms of the body flea index in 2005 were Yanchi County, Dingbian County, Wusheng County, and Otog Front Banner; in 2008, they were Lingwu city, Xingqing District, Dingbian County, and Otog Banner; and in 2010, they were Urad Middle Banner, Hanggin Banner, Jiuyuan District, and Hondlon District, in which the percentage of positive hot spot regions to the total area was 14.81%. There were three hot spot regions (Yanchi County, Dingbian County, Otog Banner) in terms of body flea index in 2011, in which the percentage of positive hot spot regions to the total area was 11.11%. There were two hot spot regions (Otog Banner, Wusheng County) in terms of the body flea index in 2012 and 2013, in which the percentage of positive hot spot regions to the total area was 7.41%. The proportion of the body flea index hot spot regions balanced comparatively from 2005 to 2009; the number reduced dramatically since 2009 until the upward trend of proportion was observed in 2013.

Plague has been found in Hanggin Banner of Ordos in the Inner Mongolia Autonomous Region since 2014, thus a new epidemic focus was recorded, and the body flea index of the Hanggin Banner in 2006, 2007, 2009, and 2010 was a hot spot region (see Fig. 4b, c, e, and f). Areas in Wusheng County, Otog Banner, Jiuyuan District, and Urad Middle Banner around Hanggin Banner had a relatively high body flea index with results that formed a spatial cluster distribution. These hot spot regions and their surrounding areas require further study. Many important hot spot regions changed in 2005, 2007, 2008, 2009, 2010, 2011, 2013, and 2014, which also requires further investigation regarding the environmental growth and decline of body fleas in these years.

## Discussion

The spatial-temporal distribution of Mongolian gerbils based on the body fleas index is proposed as a spatial statistical method for outbreak detection and prediction. Cluster, outlier, and hot spot analyses are new tools to detect aggregation of plague cases, to test the occurrence of any statistically significant clusters, and to provide advice on plague control.

After calculating the Moran’s I statistic of body fleas index on Mongolian gerbils annually, the result of the global autocorrelation analysis on the distribution of the body flea index was statistically significant for 2 years only (2005 and 2009), and the remaining years were not statistically significant.

It is likely that environmental factors contribute to the epidemic and development of the body flea index. Some studies have shown that environmental and climatic factors are related to the high incidence of body fleas. Climatic factors are also closely related to plague [24, 25]. Climate data including annual precipitation can influence the distribution of hosts and fleas as well as the population density of hosts and fleas, which then determine the geographic distribution of plague [26]. The desert steppe experiences drought nine out of ten years, and food production depends on the frequency of rainfall. The Mongolian gerbil is especially fond of plant seeds that sprout during the summer rains. Abundant rainfall during these two consecutive years forms a key element for the increasing number of the insects feeding off the Mongolian gerbil, and leading to a greater transmission of plague. Fleas are most active at temperatures between 20 and 30 degrees Celsius [27]. The fastigium of body fleas during the warmer season occurs because the number of body fleas decreases during the cold season as the temperature varies greatly between the inside and outside of the nest. The outside is very cold, so fleas do not attach on the body as they would during the warmer seasons. Only a minimal number of fleas attach to bodies of dead animals. In response to the lower temperatures, flea metabolism declines and the frequency of blood ingestion decreases, so fleas do not need to attach urgently to a body [28, 29]. The amount of captured body flea would be affected by host-trapping methodsand the survive rate of host. Diseased fleas are always found on diseased Mongolian gerbils in regions of plague epidemics. The temporal and spatial distributions of diseased fleas and diseased Mongolian gerbils are also obvious, and more diseased fleas have been found than diseased Mongolian gerbils [26].

Global Moran’s I, Anselin Local Moran’s I, and the Getis-Ord methods are useful for understanding the occurrence and location of clusters relating to body flea index data across the study area. Dingbian County presented animal plague prevalence, and Yanchi County and Otog Front Banner observed the presence of the plague as well. Mongolian gerbils have significant migration abilities, presenting the distribution trend of irregular points and distribution points of high densities on island, so the disease spreads fast, causing a wide range of localized outbreaks of serious infection. Local herdsmen have built enclosed pastures in the epidemic foci, so Mongolian gerbils have migrated from the natural distribution regions to the closed grasslands that has rich food sources, which has resulted in the formation of a high density of rats around the herdsmen’s houses. Accordingly, ectoparasite fleas on rodents have increased. The Mongolian gerbil density changed greatly in Dingbian County from 2005 to 2012 because plague among animals was prevalent in 2006. The Mongolian gerbil density obviously decreased with the plague outbreak and epidemic control; internal factors such as reproduction capacity and the external environment have played a role in this reduction, especially the influence of food production.

Vigorous growth of vegetation in the Dingbian County epidemic area has been encouraged due to a policy promoted by the Chinese government to return cultivated land to forest since 2001, but the high reproductive rate and position of Mongolian gerbils caused them to migrate, thus providing an environment for ectoparasite fleas to live on rodents or ectoparasite fleas to bite rodents. When the original desert grassland turned into meadow steppe, the density of the main host animal decreased, and thus the body flea index decreased as well.

**Fig. 5:**
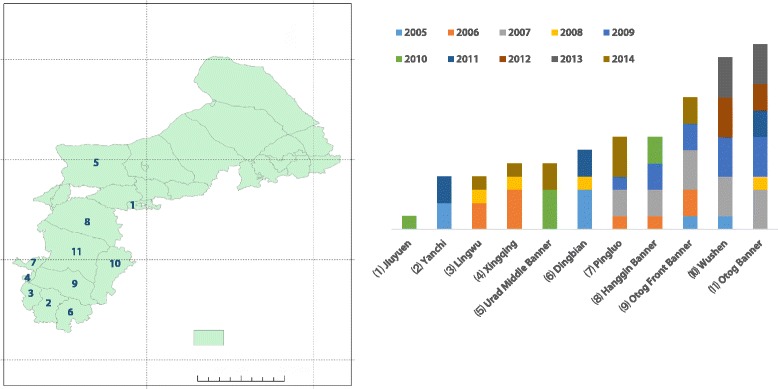
The spatial and temporal distribution of identified clusters of the body flea indexes with significant higher incidences in the natural focus of plague relating to the Mongolian gerbil, from 2005 to 2014

**Fig. 6:**
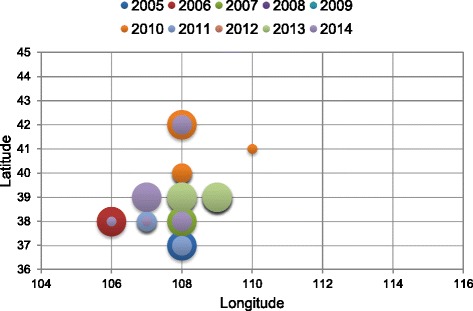
Bubble chart of gravity center for the high incidence in the southwest of the natural focus of the plague relating to the Mongolian gerbil, from 2005 to 2014

The hot spot locations in terms of body fleas have been focused in the southwest regions where Mongolian gerbil populations live (see Fig. 5 and Fig. 6). In terms of time series, the area of the epidemic focus gradually increased from 2005 to 2007, declined rapidly in 2008 and 2009, and then decreased slowly and began trending towards stability from 2009 to 2014. For the spatial change, the epidemic focus regions began moving northward from the southwest epidemic focus from 2005 to 2007, and then moved from north to south in 2007 and 2008. As Mongolian gerbils do not hibernate, the survival rate of immature Mongolian gerbils and the quantity of immature Mongolian gerbils both increased in response to improved vegetation. When Mongolian gerbil numbers increase rapidly, most of the new nests have a diluted amount of body fleas, so the exterior and interior environments are not suitable for fleas to multiply rapidly. Thus, a negative relationship between the number of Mongolian gerbils and the number of fleas was demonstrated.

The temporal and spatial distributions of the epidemic focus were affected by natural and human factors. Frequent fly ash and sandstorms affected the propagation velocity and plague transmission during the April and May of each year under study. Rainfall and dust fall have provided a migratory route for fly ash into a new location, fleas may drift and remain alive due to this and then find a new host or bite other insects. Animal plague prevalence was related to many factors, while the populations of their natural enemies such as accipiter, mustelids, and fox decreased because of human destruction of the environment. When treatment measures for epidemic outbreaks included the removal of rats and flea destruction, the rat and flea populations declined sharply in the years that followed, only to escalate into new epidemic outbreaks later on. Improved vegetation provided adequately to increase Mongolian gerbil populations. Uncultivated land is an appropriate habit for Mongolian gerbils. Vegetable seed, flax, and freshly planted wheat led to a high abundance of gerbils, and followed by an increase in flea population size.

## Conclusions

The spatial and temporal distribution of the epidemic focus determined by analyzing the distribution of the body flea indexes has shown the following: (1) The body flea index plays an important part in plague infection of the epidemic foci of Mongolian gerbils and is a vital measurement parameter for the spatial and temporal distribution of epidemic foci. (2) The epidemic had a high incidence in the southwest of the plague natural foci of the Mongolian gerbil from 2005 to 2014. (3) From the time perspective, there were changes in the distribution patterns of the body flea index in 2005, 2006, 2009, 2013, and 2014; changes in the hot spot areas of the body flea index occurred in 2005, 2007, 2008, 2009, 2010, 2011, 2013, and 2014. (4) Numerous factors contribute to the occurrence of plague, and the body flea index is influenced by seasonal variation, and human activity and natural factors. The spatial and temporal distribution of epidemic foci will change correspondingly.

## Additional file

Additional file 1: Multilingual abstract in the five official working languages of the United Nations. (PDF 763 kb)

## Abbreviations

GIS: Geographic information system
HH: High-high
HL: High-low
LH: Low-high
