# Supplementary material for: Temporal and spatial distribution characteristics in the natural plague foci of Chinese Mongolian gerbils based on spatial autocorrelation
Source: Infect Dis Poverty. 2017 Aug 7;6:124. doi: 10.1186/s40249-017-0338-7 (PMC5545858; doi:10.1186/s40249-017-0338-7)
Supplement: Additional file 1: — Multilingual abstract in the five official working languages of the United Nations. (PDF 763 kb) [file 40249_2017_338_MOESM1_ESM.pdf]

Translation of the abstract into the six official working languages of the United Nations

خصائص التوزيع الزماني والمكاني في بؤر الطاعون الطبيعي لليرابيع المنغولية الصينية استنادا إلى الترابط الذاتي المكاني

هاي-ون دو، يونج وانج، دا - فانج جوانج، شياو سان جيانج

ملخص

خلفية: مؤشر أعشاش برغوث اليربوع المنغوليMeriones unguiculatus مؤشر حاسم للوقاية من ومكافحة الطاعون، والذي يمكن استخدامه ليس فقط للكشف عن التوزيعات المكانية والزمانية لليربوع المنغوليMeriones unguiculatus، ولكن أيضا للكشف عن نهج المجموعة الخاص به. وقد كشف هذا البحث عن خصائص التوزيع الزماني والمكاني وبؤر الطاعون الطبيعية لليربوع المنغولي من قبل مؤشر برغوث الجسم من 2005 إلى 2014، من أجل التنبؤ تفشي الطاعون.

أساليب: تم استخدام الارتباط الذاتي المكاني العالمي لوصف نمط التوزيع المكاني بأكمله من مؤشر انتشار البرغوث في الجسم في بؤر الطاعون الطبيعية من اليرابيع المنغولية الصينية الشائعة. كما تم استخدام التحليل العنقودي و التحليل الخارجي و تحليل النقط الساخنة للكشف عن شدة المجموعات استنادا إلى أساليب نظام المعلومات الجغرافية. كمية البراغيث في أعشاش M. unguiculatusفي مواقع الترصد الخفرية من عام 2005 إلى عام 2014، وبيانات كثافة العائل لمنطقة الدراسة من 2005 - 2010 المستخدمة في هذه الدراسة قدمها المركز الصيني لمراقبة الأمراض والوقاية .

النتائج: ويظل الوباء يرتكز في مناطق اليرابيع المنغولية وهي نفس مناطق النقط الساخنة المتعلقة بمؤشر انتشار البرغوث في الجسم. المناطق ذات التجمعات العالية لها نمطا مماثلا لنمط مما يشير إلى أن خطر انتقال الطاعون مرتفع نسبيا. أما من حيث التسلسل الزمني، فقد ازدادت مساحة الوباء تدريجيا من 2005 إلى 2007، فقد انخفضت بسرعة في عامي 2008 و 2009، ثم انخفضت ببطء وبدأت تتجه نحو الاستقرار في الفترة من 2009 إلى 2014. وبالنسبة للتغير المكاني، بدأت مناطق الوباء تتحرك شمالا من التركيز الوبائي الجنوبي الغربي لليرابيع المنغولية في الفترة من 2005 إلى 2007، ثم انتقلت من الشمال إلى الجنوب في عامي 2007 و 2008.

الاستنتاجات: يكشف مؤشر انتشار البرغوث في الجسم لبؤر اليرابيع الصينية عن خصائص تجميع مكانية وزمانية هامة من خلال استخدام الترابط الذاتي المكاني. ويتأثر تنوع التوزيع المؤقت والمكاني أساسا بالتغيرات الموسمية والنشاط البشري والعوامل الطبيعية.

Translated from English version into Arabic by Mahmoud Sami, through

TRANSLATORS
WITHOUT BORDERS

基于 GIS 空间自相关的中国长爪沙鼠疫源地时空分布特征

杜海文，王勇，庄大方，姜小三

摘要

引言：长爪沙鼠巢蚤指数是预防和控制鼠疫的关键指标，不仅可用于探测长爪沙鼠的时空分布，而且能够揭示其聚集规律。本研究通过检测 2005-2014 年长爪沙鼠疫源地鼠体蚤指数确定的时空分布特征，根据鼠体蚤随长爪沙鼠的活动而形成的空间分布，来预测鼠疫的爆发。

方法：运用时空分析方法对 2005-2014 年长爪沙鼠疫源地鼠体蚤数据进行全局自相关、聚类和异常值分析和热点分析。在本研究中使用的 2005-2014 年长爪沙鼠巢蚤监测点观测数量和 2005-2010 年的宿主密度数据由中国疾控中心提供。

结果：长爪沙鼠的疫源地与鼠体蚤指数热点区域保持一致。高值聚类是鼠体蚤指数的主要分布模式，即鼠体蚤指数比较高，从时间序列上来看，鼠体蚤指数热点区域数量在 2005-2007 年呈逐渐上升趋势、2008-2009 年急速下降、2009-2014 年缓慢降低后趋于平稳。从空间变化规律来看，鼠

体蚤指数热点区域在 2005-2007 年从长爪沙鼠疫源地的西南角向北移动、2007-2008 年由北向南移动。
结论：采用空间自相关分析中国鼠体蚤指数显示出显著的空间和空间聚集特征。时空分布的多样性主要受季节变化、人类活动和自然因素的影响。

Translated from English version into Chinese by Hai-Wen Du, Yong Wang

Caractéristiques de répartition temporelle et spatiale dans les foyers de peste naturels des gerbilles sino-mongoles basé sur l'autocorrélation spatiale

Hai-Wen Du, Yong Wang, Da-Fang Zhuang, Xiao-San Jiang

Abstrait
Contexte: L'indice des puces de nids du *Meriones unguiculatus* est un indicateur critique pour la prévention et le contrôle de la peste, qui peut être utilisé non seulement pour détecter les répartitions spatiales et temporelles du *Meriones unguiculatus*, mais également pour révéler ses règles de groupement. Cette recherche a détecté les caractéristiques de répartition temporelle et spatiale des foyers de peste naturels des Gerbilles de Mongolie par indice de puces corporelles de 2005 à 2014, afin de prévoir les épidémies de peste.
Méthodes: L'autocorrélation spatiale globale a été utilisée pour décrire l'ensemble du modèle de répartition spatiale de l'indice de puces corporelles dans les foyers de peste naturels des gerbilles sino-mongoles types. L'analyse de groupement et de cas déviant et l'analyse des endroits à risque ont également été utilisées pour détecter l'intensité des groupements selon les méthodes de système d'information géographique. La quantité de puces de nid du *M. unguiculatus* dans les sites de surveillance sentinelles de 2005 à 2014 et les données sur la densité d'hôtes du champ d'étude de 2005 à 2010, utilisées lors de la présente étude, ont été fournies par le Centre chinois pour le contrôle et la prévention des maladies.
Résultats: Les régions cibles sujettes aux épidémies des Gerbilles de Mongolie restent les mêmes que les régions à risque liées à l'indice des puces corporelles. Les régions à groupements élevés possèdent une configuration similaire à celle de l'indice de répartition des puces corporelles indiquant que le risque de transmission de la peste est relativement élevé. En termes de séries chronologiques, la région cible sujette à l'épidémie a progressivement augmenté de 2005 à 2007, diminué rapidement en 2008 et 2009, puis diminué lentement et commencé à progresser vers la stabilité de 2009 à 2014. Pour le changement spatial, les régions cibles sujettes à l'épidémie ont commencé à se déplacer vers le nord de l'épidémie du sud-ouest des Gerbilles de Mongolie de 2005 à 2007, puis se sont déplacées du nord au sud en 2007 et 2008.
Conclusions: L'indice de puces corporelles des foyers de gerbilles chinois révèle d'importantes caractéristiques d'agrégation spatiale et temporelle à travers l'utilisation de l'autocorrélation spatiale. La diversité de la répartition temporaire et spatiale est principalement affectée par la variation saisonnière, l'activité humaine et les facteurs naturels.

Translated from English version into French by Sabyna Delperdange, through

Характеристики временного и пространственного распределения в природных очагах чумы в районах обитания китайских и монгольских песчанок на основе пространственной автокорреляции

Хай-Вэнь Ду, Юн Ван, Да-Фан Цжуан, Сяо-Сань Цзян

Аннотация

Обоснование исследования. Индекс обилия блох в гнёздах *Meriones unguiculatus* является важным показателем для профилактики и борьбы с чумой. Он может использоваться не только для определения пространственного и временного распределения *Meriones unguiculatus*, но и для выявления правила их группирования. В данном исследовании были определены временные и пространственные характеристики природных очагов чумы, переносимой монгольскими песчанками, в зависимости от индекса обилия блох на теле с 2005 по 2014 годыдля того, чтобы предсказывать вспышки этого заболевания.

Методы. Для описания полного пространственного распределения индекса обилия блох на телах типичных китайских и монгольских песчанок в природных очагах чумы был применён метод глобальной пространственной автокорреляции. Для определения интенсивности кластеров на основе географических информационных систем также использовались анализ кластеров и выбросов и анализ "горячих точек". Данные о количествах гнёзд блох у *M. unguiculatus* в местах дозорного эпиднадзора с 2005 по 2014 год и о плотности распределения носителя инфекции в районе проведения исследования в 2005-2010 годы, использованные в данном исследовании, были предоставлены Китайским центром по борьбе и профилактике болезней.

Результаты. В отношении индекса обилия блох эпидемические очаги в местах обитания монгольской песчанки соответствуют районам "горячих точек". Для мест с высокой кластеризацией характерно аналогичное распределение индекса обилия блох, index что указывает на относительно высокий риск передачи чумы. Что касается временных рядов, то область эпидемического очага постепенно увеличилась с 2005 по 2007 год, резко сократилась в 2008 и 2009 годах и затем начала стабилизироваться с 2009 по 2014 год. В части пространственных изменений районы эпидемических очагов смещались к северу от юго-западных мест обитания монгольских песчанок с 2005 по 2007 год и затем начали двигаться с севера на юг в 2007 и 2008 годах.

Выводы. С помощью пространственной автокорреляции индекс обилия блох на телах китайских песчанок в очагах выявил важные пространственные и временные характеристики группирования. На неоднородность временного и пространственного распределения главным образом влияют сезонные колебания, деятельность человека и природные факторы.

Translated from English version into Russian by Natalia Potashnik, through

Características de la distribución espacial y temporal de los focos naturales de peste de jerbos de Mongolia, China basada en la autocorrelación espacial

Hai Wen Du, Yong Wang, Da-Fang Zhuang, Xiao-San Jiang

Resumen

Antecedentes: El índice de pulgas nido de *Meriones unguiculatus* es un indicador clave para la prevención y control de la peste, que puede utilizarse no solo para detectar las distribuciones espaciales y temporales de *Meriones unguiculatus*, sino también para revelar el cluster. Esta investigación detectó las características de distribución espacial y temporal de los focos naturales de peste de jerbos de Mongolia por el índice de pulgas del cuerpo de 2005 a 2014, para predecir los brotes de peste.

Métodos: Se utilizó la autocorrelación espacial global para mostrar el patrón de distribución espacial del índice de pulgas de cuerpo en los focos naturales de peste de jerbos típicos de Mongolia, China. También se analizó el cluster, los casos aislados y los focos para detectar la intensidad de los clusters basados en métodos de sistema de información geográfica. La cantidad de *M. unguiculatus* nidos de pulgas en los sitios de vigilancia centinela de 2005 a 2014 y los datos de densidad de huésped de la zona de estudio entre 2005 y 2010 utilizados en este estudio fueron proporcionados por el Centro de China para la Prevención y el Control de Enfermedades.

Resultados: Las zonas de foco epidémico de los jerbos de Mongolia son las mismas que los focos de pulgas de cuerpo. Las áreas de cluster alto poseen un patrón similar al patrón de distribución de la pulga de cuerpo índice que muestra que el riesgo de transmisión de la peste es relativamente alto. En términos de series temporales, el área del foco epidémico gradualmente aumentó de 2005 a 2007, disminuyó rápidamente en 2008 y 2009, y luego disminuyó lentamente y tendió a la estabilidad de 2009 a 2014. En cuanto al cambio espacial, los focos de epidemia de los jerbos de Mongolia comenzaron a moverse hacia el norte desde el sudoeste de 2005 a 2007 y luego de norte a sur en 2007 y 2008.

CONCLUSIONES: El índice de focos de pulgas en el cuerpo de los jerbos de China revela características importantes de agregación espacial y temporal mediante el empleo de autocorrelación espacial. La diversidad de la distribución espacial y temporal depende de la estacionalidad, la actividad humana y los factores naturales.

Translated from English version into Spanish by Amparo Muñoz, through
